# What is this duplicate publication thing?

**DOI:** 10.1093/jhps/hnv065

**Published:** 2015-10-26

**Authors:** Richard Villar

**Affiliations:** Editor-in-Chief, Journal of Hip Preservation Surgery

One of the commonest queries I receive as Editor-in-Chief is whether a submission can be accepted when the author’s message is already known. The paper may have been presented at meetings a couple of times and might even have appeared in abstract form. Can we, as a journal, go ahead and publish without eyebrows being raised and the accusation of duplicate publication being made?

The story starts with a classic of the editing world. His name was Franz Ingelfinger, a German-American, and for 9 years (1967–76) he was the Editor of the *New England Journal of Medicine*. You may have heard of him, although on the other hand you may not.

Ingelfinger [1], in 1969, proposed a key rule of the publication game by which we all adhere to this day. He laid out the definition of what we now call duplicate (or dual) publication. This is when the same material is published more than once by either the author or the publisher. It is not the same as plagiarism, which is when someone different performs republication [2]. Duplicate publication is more common than you think. Have a look at Déjà vu [3], a database of highly similar citations, where you will see the problem laid out before you. Duplicate publication does certainly exist, is regarded as a form of research misconduct, and is something that as an editor you need always to keep in the back of your mind.

I can see the pressure, of course. Journals take time to reach a decision, so why not submit the same paper to two, three, maybe four or five journals simultaneously? After all, is that not what creative authors do in the big, bad world of mass-market writing? Then before you know it, two of your identical submissions are accepted, one some months after the other and … bingo! … you have entered the ranks of the duplicate publishing author.

Or, you may be a particularly prolific author and have forgotten that one of your team submitted a paper on a specific topic 2 years earlier. It may have been published in some lesser-known journal and may even have passed you by. It happens. A subsequent trainee has a go, unaware of his predecessor’s actions and out comes that second paper. Again, unwittingly you have become a duplicate author and it is not a happy place to be.

Anyway, why should a journal worry? Why should anyone be concerned? After all, each of us seeks to disseminate our work as widely and as rapidly as we can. Yet the publishing dilemma is clear because duplicate publications can cause trouble for a number of reasons [4]:

1. They waste finite resources. Journals have a limited number of pages available and duplicate submissions will be reviewed twice, indexed twice, copyedited twice, distributed twice and so on.
2. They overload available medical information. It simply takes longer to find what you need.
3. They overemphasize the findings. The classic is a meta-analysis looking at the antiemetic efficacy of ondansetron [5]. This showed that duplicate publication led to an overestimation of ondansetron’s efficacy by 23%.
4. Duplicate publications contravene copyright law, if you have signed your copyright across to another journal. This is less of a problem these days with Open Access, where the requirement for an author to hand over copyright is becoming rare.

Language bias is also an issue, especially for an international journal and certainly when it comes to the influence on meta-analyses. One study [6] assessed whether authors were more likely to publish positive results in English language journals rather than German. The answer was a resounding ‘yes’. However, in the process, the authors had to exclude 19 of their 62 studies (31%) because they were duplicate publications, one in English and the other in German. That is a huge number.

The Anglo-German study may be exceptional but the problem is manifestly global. In the former *Journal of Bone and Joint Surgery (Br)* a study of so-called original articles in that journal showed one in 13 to be duplicate or fragmented publications [7]. Fragmented publishing is the same as so-called salami-slicing. A more recent paper from South Korea in 2008 [8] looked specifically at publications in Korean Medical Journals indexed in KoreaMed and found 27 of 455 (5.93%) articles were duplicates.

On Medline presently, there sit 22 million references [9] to journal articles in the life sciences, predominantly biomedicine. Just think how much space there might be for the rest of us if the duplicates were removed? There is clearly a problem out there and each of us—authors, reviewers and editors—needs to be on our guard.

So what are the rules of the game? These are well described by the International Committee of Medical Journal Editors (ICMJE) and followed by many [10]. The guidelines look at four things: duplicate submission, duplicate publication, acceptable secondary publication and manuscripts based on the same database. I would suggest reading them in full. There is nothing surprising about any of them.

Two things are very clear, however. First, submitting the same paper to two journals simultaneously is a non-starter. Second, if your submission is very similar to work reported elsewhere then (i) reference that work and (ii) say so in your letter of submission. However, if your work has appeared as a presentation or abstract before, that does not matter, but do reference it anyway.

Of course, none of what I have written here is applicable to *JHPS*, but duplicate publication is certainly a topic about which I am asked repeatedly by potential authors. That need for us each to publish frequently and widely seems insatiable. So please have a read of the ICMJE guidelines [10] and, if your submission fits, by all means send it in.

Now turning to *JHPS* and its content, which in many respects is why I am here, what specifically grabbed my attention in the last issue (2.2)? Naturally every paper we publish has done well to appear on our pages and each has added its own aliquot of information to the ever-expanding field of hip preservation surgery. However, I was especially caught by Kalisvaart and Safran’s [11] review on hip microinstability, as this is manifestly an area of hip surgery that is expanding fast. Watch that space, for sure. I was also incredibly impressed by the minisymposium on evolving concepts in extra-articular hip pathology [12–16], put together by some really supportive authors and with Hal Martin’s truly dedicated lead. I recommended it last time and I do it again now. Do read the whole minisymposium; in fact do please peruse every last paper published. You will not be disappointed.

And for this issue? Issue 2.3? Oh boy, things are beginning to take off at such a pace and in a way that I for one never expected so early in the life of a fledgling journal. Downloads are skyrocketing and our submission inbox always, always, always contains something interesting. Actually, everything sent in is interesting. Why, oh why can we not publish the lot?

It is our reviewers, you see. They have an eye for the uncanny, the worthwhile, the message we can all take home. They are a tremendous lot and deserve the largest vote of thanks any editor can give.

So when you look at issue 2.3 do be sure to read everything, although I confess to lingering a little longer over two papers in particular. The first was the excellent review by Bardakos [17] on all those impingements that are not femoroacetabular. There are plenty of them out there. The other was that wonderful paper by Cvetanovich *et al.*[18] on hip arthroscopy after Bernese periacetabular osteotomy. I loved that one, not only for its content but also for its subject matter. Those of us who have been around for longer than most well remember the days when osteotomists and arthroscopists would rarely speak. Now look at it. There are papers being submitted from groups that offer both. The true friendship that hip preservation has now engendered is there for us all to see.

My very best wishes to you all.

## References

[1] Ingelfinger FJ Definition of “sole contribution”. NEJM 1969; 281: 676–77. 5807917 10.1056/NEJM196909182811208

[2] Available at:https://en.wikipedia.org/wiki/Duplicate_publication. Accessed: 30 August 2015.

[3] Available at:http://dejavu.vbi.vt.edu/dejavu/. Accessed: 30 August 2015.

[4] Abraham P Duplicate and salami publications (editorial). J Postgrad Med 2000; 46: 67–9. 11013467

[5] Tramèr MR Reynolds AJM Moore RA Impact of covert duplicate publication on meta-analysis: a case study. BMJ 1997; 315: 635–40 9310564 10.1136/bmj.315.7109.635 PMC2127450

[6] Egger M Zellweger-Zähner T Schneider M Language bias in randomized controlled trials published in English and in German. Lancet 1997; 350: 326–9. 9251637 10.1016/S0140-6736(97)02419-7

[7] Gwilym SE Swan MC Giele H One in 13 ‘original’ articles in the Journal of Bone and Joint Surgery are duplicate or fragmented publications. J Bone Joint Surg 2004; 86B: 743–5. 10.1302/0301-620x.86b5.14725 15274274

[8] Kim SY Hahm CK Bae C-W Duplicate publications in Korean Medical Journals indexed in KoreaMed. J Korean Med Sci 2008; 23: 131–3. 18303213 10.3346/jkms.2008.23.1.131 PMC2526492

[9] Available at:http://www.nlm.nih.gov/pubs/factsheets/medline.html. Accessed: 30 August 2015.

[10] Available at:http://www.nlm.nih.gov/pubs/factsheets/medline.htmlhttp://www.icmje.org/recommendations/browse/publishing-and-editorial-issues/overlapping-publications.html. Accessed: 30 August 2015.

[11] Kalisvaart MM Safran MR Microsinstability of the hip—it does exist: etiology, diagnosis and treatment. J Hip Preserv Surg 2015; 2: 123–35. 27011829 10.1093/jhps/hnv017 PMC4718498

[12] Martin HD Evolving concepts in extra-articular hip pathology. J Hip Preserv Surg 2015; 2: 91. 27011824 10.1093/jhps/hnv018 PMC4718502

[13] Lemos N Possover M Laparoscopic approach to intrapelvic nerve entrapments. J Hip Preserv Surg 2015; 2: 92–8. 27011825 10.1093/jhps/hnv030 PMC4718483

[14] Martin HL Reddy M Gómez-Hoyos J Deep gluteal syndrome. J Hip Preserv Surg 2015; 2: 99–107. 27011826 10.1093/jhps/hnv029 PMC4718497

[15] Matsuda DK Matsuda NA Endoscopic hip osteotomies: less invasive approaches to peri-acetabular, proximal femoral and pubic symphyseal procedures. J Hip Preserv Surg 2015; 2: 108–15. 27011827 10.1093/jhps/hnv025 PMC4718487

[16] Guanche CA, Hamstring injuries. J Hip Preserv Surg 2015; 2: 116–22. 27011828 10.1093/jhps/hnv026 PMC4718494

[17] Bardakos NV Hip impingement: beyond femoroacetabular. J Hip Preserv Surg 2015: 2: 2.3. 10.1093/jhps/hnv049 PMC4765300 27011843

[18] Cvetanovich GL Heyworth BE Murray K Hip arthroscopy in patients with recurrent pain following Bernese periacetabular osteotomy for acetabular dysplasia: operative findings and clinical outcomes. J Hip Preserv Surg 2015; 2: 2.3. 10.1093/jhps/hnv037 PMC4765306 27011852

